# Pattern of irritable bowel syndrome and its impact on quality of life in primary health care center attendees, Suez governorate, Egypt

**DOI:** 10.4314/pamj.v9i1.71177

**Published:** 2011-05-18

**Authors:** Ahmed Abdulmajeed, Mohamed A Rabab, Hamdy A Sliem, Nour Eldein Hebatallah

**Affiliations:** 1 Department of Family Medicine, Faculty of Medicine, Suez Canal University, Egypt; 2 Department of Internal Medicine, Faculty of Medicine, Suez Canal University, Egypt

**Keywords:** Irritable bowel syndrome, quality of life, Roma II

## Abstract

**Introduction:**

Irritable bowel syndrome (IBS) is one of the most common disorders diagnosed by gastroenterologists and a common cause of general practice visits. Although this disease is not life threatening, patients with IBS seem to be seriously affected in their everyday life. The study was designed to explore the pattern of IBS in clinical practice and the impact on the quality of life.

**Methods:**

This is a case control descriptive study. 117 individuals were included in this study. Rome II criteria were used for the diagnosis of IBS. Impact of IBS on patient's quality of life was determined by irritable bowel syndrome quality of life (IBS-QOL) questionnaire.

**Results:**

Prevalence of IBS among the study sample was 34.2%. 10% were IBS-Diarrhea, 37.5% were IBS-Constipation and 52.5% were alternators. There is statistical insignificant relationship between IBS (+) and age while it was a significant relation regarding gender (more common among women 80%). There is statistical significance relationship between IBS (+) on one hand and marital status and occupational status on the other hand. Patients with IBS had statistically significant lower scores for all IBS- QOL domains compared with the control group.

**Conclusion:**

IBS is a prevalent disorder that affects females more than males and it has significant impacts on work, lifestyle and social well-being.

## Introduction

Irritable bowel syndrome (IBS) is one of the most common disorders diagnosed by gastroenterologists and a common cause of general practice visits [1]. It is widespread in all societies and socio-economic groups [2]. It is a common health problem affecting a substantial proportion of the population. Prevalence estimates usually range from 12–30% with rates vary significantly between countries and depend on the diagnostic criteria used [3]. American College of Gastroenterology IBS Task Force defined IBS as abdominal pain or discomfort that occurs in association with altered bowel habits over a period of at least 3 months [4]. Altered bowel function, with the predominant bowel symptom determining the sub-classification of IBS: IBS with constipation (IBS-C), IBS with diarrhea (IBS-D), or IBS with alternating symptoms of constipation or diarrhea (IBS-A) [5]. Doctors generally rely on symptom-based criteria. At least three sets of diagnostic criteria have been developed, including the Manning, Rome I and Rome II criteria. The Rome I and II criteria are more refined than the Manning criteria [6]. The Rome III updated criteria were published in April 2006. The principle difference between Rome III guidelines as compared with the Rome II criteria lies in the less restrictive timeframe for symptoms. Whereas the Rome II criteria require symptoms to be present for at least 12 weeks (not necessarily consecutive) in the past 12 months, the Rome III criteria require symptoms to originate for 6 months prior to diagnosis, and be currently active for 3 months [7]. Many IBS patients have psychological symptoms including depression, anxiety, tension, insomnia, frustration. The onset and course of IBS are strongly influenced by psychosocial factors. However, IBS-D and IBS-C were both associated only with high anxiety, but not depression, compared to the non-IBS control group [8]

In assessing the impact of a chronic disease such as IBS on sense of wellbeing and daily functioning, patient-centered outcome data of Health Related Quality of Life (HRQOL) are essential. Previous studies of the impact of IBS on quality of life have either used generic health-related quality of life measurements, such as SF-36, or IBS-specific HRQOL instruments. Disease-specific measures are especially used in clinical trials, while generic HRQOL measures are designed to evaluate aspects that are applicable to a population and therefore can provide a basis for comparisons with data from the general population [2]. Although this disease is not life threatening, patients with IBS seem to be seriously affected in their everyday life [9]. They describe their HRQOL as similar to or worse than that of patients with more serious or life-threatening illnesses such as gastroesophageal reflux disease (GERD), asthma, and end-stage renal disease [10]. For these reasons, medical and regulatory Food and Drug Administration (FDA) recommend routine assessment of HRQOL of more severely affected patients [11]. In the United States, IBS also puts a heavy economic burden on patients, employers, and the healthcare system, resulting in more than $10 billion in direct costs (eg, from office visits, medications) and $20 billion in indirect costs (eg, through work absenteeism and reduced productivity) each year[12].

Despite increase awareness of functional bowel disease as the irritable bowel syndrome, there is no accurate information on the epidemiology of it in Egypt. However, the irritable bowel syndrome is believed to be very common.

This study aims to improve quality of life of patients with irritable bowel syndrome with 2 objectives; to assess the prevalence and symptom patterns of irritable bowel syndrome and to assess the impact of irritable bowel syndrome on the quality of life.

## Methods

The study was carried out at 24 October Family Health Center, which locate at an urban area in Suez governorate from January 2008 to August 2009. Subjects were individuals fulfilling the inclusion criteria as both male & female gender aged 18 to 65 years. All visited the center for general medical problems. The exclusion criteria included previously diagnosed patients with Crohn′s disease, ulcerative colitis, Coeliac disease, diverticulitis, and peptic ulcer. The sample size was calculated using EPI-INFO statistical program with help of the following information: expected frequency=20%, worst acceptable result = 30%, so the sample size = 106. If we expect a 10% drop-out rate, then the sample size =117.

A validated questionnaire was used in collecting data through face to face interview for eligible patients. The questionnaire was divided into three parts: The first part was designed to investigate individuals′ socio-economic demographic data and patient characteristics as age, sex, marital status, education, occupation, three regular meals per day, fiber consumption, daily tea consumption, daily coffee consumption, daily cola consumption, smoking, laxative use, previous abdominal operation, previous psychological stress, and relation to hemorrhoids [13]. The second part was Rome II criteria for the diagnosis IBS & supportive symptoms for more detailed symptom description. Patients were defined as having IBS with diarrhea (IBS-D) if they experienced one or more of the following: Looser/more watery stools than usual, the need to pass stools more often than usual (>3 times/day), or periods of urgency. Patients with other bowel habit patterns were defined as having alternating IBS (IBS-A). Differentiation into these specific IBS subtypes was based only on the symptoms suffered by each respondent, without reference to a specific time frame [6].

The third part was designed to investigate the impact of irritable bowel syndrome on quality of life that was determined by irritable bowel syndrome quality of life (IBS-QOL) questionnaire (34 items) [14]. We used (IBS-QOL) questionnaire to compare between patients with IBS, according to the Rome II criteria (cases), and those not meeting the Rome criteria (control). The individual responses to the 34 items were summed and averaged for a total score and then transformed to a 0-100 scale for ease of interpretation with higher scores indicating better IBS specific quality of life. Eight subscale scores for the IBS-QOL were: 1-Dysphoria, 2-Interference with Activity, 3-Body Image, 4-Health Worry, 5-Food Avoidance, 6-Social Reaction, 7-Sexual concerns, 8-Relationships. Statistical analysis was performed by using the Epi- Info (EPI6) computer software statistical package.

### Ethical considerations

Aim of the research was explained to the participants. Informed consent from the participants was taken before starting the interview. The study was approved by the ethics committee of faculty of medicine, Suez Canal University and has been performed in accordance with the ethical standards laid down in the 1964 Declaration of Helsinki.

## Results

Out of 117 individuals, 40 adults had IBS (34.2%). 52.5% of them had IBS-A, 37.5% had IBS-C and 10% had IBS-D. Table 1 Shows that the majority of IBS (+) 48.72% were in age group 40-49 years old and there was no statistically significant relationship between age and IBS (+) (P>0.05). The prevalence of IBS (+) was significantly higher in females 41.1% with statistical difference with males (P<0.05). IBS (+) were highest in widow group 80% with statistical significance relationship between IBS (+) and marital status (p<0.05). There was no statistical significant relationship between IBS (+) and educational status (P>0.05). IBS (+) was highest in house-wives group 51.5%, there was statistical significant relationship between IBS (+) and occupational status (P<0.05). Table 2 shows characteristics of respondents with and without IBS-type symptoms. There was no statistical significant relationship between IBS (+) and persons not eating three regular meals per day, subjects without fiber in their diet, daily tea, coffee or cola consumption, and smoking (P>0.05). There was a significant relationship between IBS and psychological stress (p<0.05). There was no significant relationship between IBS and laxative Use, previous abdominal operation or hemorrhoid presence (p>0.05).

**Table 1:** Distribution of irritable bowel syndrome (IBS+) subjects according to different socio-demographic characteristics in primary health care center attendees, Suez governorate, Egypt

Item	IBS							
	+ (N=40)		- (N=77)		Total (N=117)		X^2^	P

	N	%	N	%	N	%		
**Age**								
18–29 years	14	28.57	35	71.43	49	41.88		
30–39	5	22.73	17	77.27	22	18.81		
40–49	19	48.72	20	51.28	39	33.33	5.73	0.125
≥ 50	2	28.57	5	71.43	7	5.98		
**Gender**								
Male	8	20.6	31	79.4	39	33.3		
Female	32	41.1	46	58.9	78	66.7	4.8	0.028*
**Marital status**								
Single	8	20.6	31	79.4	39	33.33		
Married	28	38.4	45	61.6	73	62.39		
Widow	4	80	1	20	5	4.28	8.4	0.014*
Divorced	0	0	0	0	0	0		
**Educational status**								
Illiterate	0	0	2	100	2	1.72		
Write & read	3	33.3	6	66.7	9	7.69		
Moderate	27	41.5	38	58.5	65	55.55		
High	10	24.3	31	75.7	41	35.04	4.3	0.2
**Occupational status**								
Officers	12	31.5	26	68.5	38	32.48		
Workers	4	23.5	13	76.5	17	14.53		
Housewives	18	51.5	17	48.5	35	29.91		
Students	0	0	13	100	13	11.11		
							13.75	0.017
Others	6	42.8	8	57.2	14	11.97		

N=number of cases +: with IBS -: without IBS *: P<0.05

**Table 2:** Characteristics of respondents with and without Irritable Bowel Syndrome (IBS)-type symptoms in primary health care center attendees, Suez governorate, Egypt

		IBS							
Item	state	+ (N=40)		- (N=77)		Total (N=117)		X^2^	P
				
		N	%	N	%	N	%		
Three regular meals/day	Present	19	47.5	37	48.1	56	47.8	0.0	0.95
	Absent	21	52.5	40	51.9	61	52.1		
									
Daily fiber consumption	Present	20	50	28	36.4	48	41.2	2.01	
	Absent	20	50	49	63.6	69	58.9		0.15
									
Daily tea consumption	0 cups	1	2.5	6	7.8	7	5.98		
	1–2 cups	31	77.5	51	66.2	82	70.1	2.1	0.34
	3+ cups	8	20	20	25.9	28	23.9		
									
Daily coffee consumption	0 cups	20	50	47	61.1	67	57.2		
	1–2 cups	20	50	29	37.7	49	41.9	2.04	0.36
	3+ cups	0	0	1	1.2	1	0.85		
									
Daily cola consumption	0 cups	24	60	45	58.4	69	58.97		
	1–2 cups	15	37.5	27	34.1	42	35.9	0.87	0.64
	3+ cups	1	2.5	5	6.5	6	5.1		
									
Smoking	Present	6	15	12	15.6	18	15.4		0.93
	Absent	34	85	65	84.4	99	84.6	0.01	
									
Laxative Use	Present	7	17.5	12	15.6	19	16.2	0.07	0.79
	Absent	33	82.5	65	84.4	98	83.8		
									
Previous abdominal operation	Present	12	30	19	24.7	31	26.5	0.38	0.53
	Absent	28	70	58	75.3	86	73.5		
									
Psychological stress	Present	33	82.5	42	54.5	75	64.1	8.86	0.002
	Absent	7	17.5	35	45.5	42	38.9		
									
Hemorrhoids	Present	13	32.5	15	19.5	28	23.9	2.43	0.11
	Absent	27	67.5	62	50.5	89	76.1		

N=number of cases +: with IBS -: without IBS

Table 3 shows the symptom patterns experienced at evaluation, such as the frequency of motions, Stool consistency and abdominal discomfort. Bowel movements < 3 times a week was 82.5% in IBS (+) patients, while in IBS (-) was 19.5%. Bowel movements > 3 times a day was 57.5% in IBS (+) patients while was 3.9% in IBS (-).Stool consistency, hard stool was 80% in IBS (+) while was 40.26% in IBS (-) and Loose stool was 52.5% in IBS (+) while was 0% in IBS (-). Straining was 80% in IBS (+) while was 5.2% in IBS (-).Urgency was 55% in IBS (+).Incomplete bowel movement was 87.5% related to IBS (+).Passing mucus was 32.5% in IBS (+)while was 0% in IBS (-). Abdominal distension was 90% in IBS (+) while was 49.35% in IBS (-).

**Table 3:** Supportive symptoms of Irritable Bowel Syndrome in a group of primary health care center attendees, Suez governorate, Egypt

	IBS							
					
	+ (N=40)		- (N=77)		Total (N=117)		X2	P-value
					
Symptoms	No	%	No	%	No	%		
Bowel movements<3 times a week	33	82.5	15	19.5	48	41.02	42.84	<0.001*
Bowel movements>3 times a day	23	57.5	3	3.9	26	22.22	43.39	<0.001*
Hard stool	32	80	31	40.26	63	53.8	16.59	<0.004*
Loose stool	21	52.5	0	0	21	17.9	48.85	<0.001*
Straining	32	80	4	5.2	36	30.76	68.56	<0.001*
Urgency	22	55	2	2.6	24	20.5	43.96	<0.001*
Incomplete bowel movement	35	87.5	2	2.6	37	31.6	87.01	<0.001*
Passing mucus	13	32.5	0	0	13	11.11	27.91	<0.001*
Abdominal distention	36	90	38	49.35	74	63.24	18.55	<0.001*

N=number of cases +: with IBS -: without IBS *: P<0.05

Table 4 shows Patients with IBS had statistically significant lower scores for all IBS- QOL domains compared with the general population (P<0.01). The most affected domain was the Food Avoidance scale and the least affected domains those of Relationships, Sexual concerns and Social reaction. By the logistic regression, daily cola consumption was unfortunately affecting QOL positively. IBS, Previous psychological stress, hard stool, straining, passing mucus and abdominal distension were negativity affecting QOL.

**Table 4:** Comparison of health-related quality of life (IBS-QOL) between respondents with and without Irritable Bowel Syndrome in a group of primary health care center attendees, Suez governorate, Egypt

Quality of life subscales Scores	IBS (+) N=40		IBS (-) N=77		t-test	P-value
				
	Mean	SD	Mean	SD		
Dysphoria	49.45	26.63	89.33	15.38	8.743	<0.001*
Interference with activity	48.66	25.17	85.81	15.42	8.538	<0.001*
Body image	61.56	23.02	92.86	12.07	8.043	<0.001*
Health Worry	53.13	28.48	91.34	15.62	7.894	<0.001*
Food avoidance	38.54	27.33	81.60	21.69	8.651	<0.001*
Social reaction	65.63	20.61	90.83	16.18	6.732	<0.001*
Sexual concerns	67.50	25.76	93.67	12.60	6.060	<0.001*
Relationships	69.79	20.30	92.86	13.16	6.511	<0.001*
Total	56.78	19.08	89.79	12.99	9.821	<0.001*

N=number of cases +: with IBS -: without IBS *: P<0.05

## Discussion

In the present study, IBS prevalence of 34.2% was found. This prevalence is different from other studies. A Canadian sample reported a prevalence of 13.5% using Rome I and 12.1% using Rome II Criteria [15]. In the US householder survey, 11% of those surveyed reported symptoms consistent with IBS [16]. In a large survey (>41 000 patients) recently conducted in eight European countries, overall prevalence of IBS was 9.6%, and ranged from 6.2 to 12% across countries [17]. Clearly, IBS prevalence can vary substantially depending on the diagnostic criteria employed and type of the study [18].

Most IBS sufferers in this study had alternating symptoms of constipation and diarrhea. This finding is consistent with Hungin et al. that reported most IBS sufferers (74%) had alternating symptoms of constipation and diarrhea as defined by doctors and diagnostic criteria [19]. Other published data reported most IBS sufferers had diarrhea predominant symptoms (IBS-D), Smith et al. found that a prevalence rate of IBS-D is the most common symptoms followed by IBS-A [20].

The current study revealed that there was no statistical significant difference between IBS+ and controls regarding age difference (p>0.05), but the IBS prevalence was higher in age group 40–49 years (48.72%). Hungin et al. found that prevalence rates were highest among those aged 25–54 years [19. Kay et al [21] and Karaman et al [13] found an inverse relationship between age and IBS prevalence, perhaps because individuals might ignore IBS-related symptoms as their organic diseases become more dominant with increased age. We found also the prevalence of IBS was significantly more common among women (41.1%) than men (20.6%). This finding is consistent with Hungin et al 19 and Andrews et al [22] that found the prevalence of IBS among women was approximately two times higher than that recorded for men in individuals diagnosed with IBS. Although, this finding is in contrast with Minnesota study demonstrated that the age-adjusted prevalence of IBS using the Rome II criteria was higher in men than in women [23]. Moreover, studies conducted in Asian countries, such as India [24] and Taiwan [25] have reported a higher male or equal gender prevalence of IBS. Cultural factors as well as study methodology may explain in part these gender differences. For example The Manning criteria have been reported to be more sensitive in diagnosing IBS in women than in men [26] and it has been suggested that the Rome criteria are less sensitive in men as well, possibly because men report fewer non-painful IBS symptoms than women [27]. In this study, the prevalence of IBS was highest in widow individuals (80%) this may be due to increasing responsibilities and stressors. Andrews et al. found prevalence was higher among unmarried individuals compared with married (7.7% vs. 5.9%) [22]. However, we can not detect any significant difference according to educational status, while there was a significant relation with occupational status. These findings are consistent with Karaman et al. that found IBS prevalence did not show any significant difference according to educational status but it was significant regarding the occupational distribution [13]. Food intolerance has been proposed as a potential cause of GI symptoms in some patients with IBS however this link is not well established. Although some patients associate onset of IBS symptoms with ingestion of particular foods identification of true food intolerance is challenging and elimination diets are typically time-consuming and difficult to implement [28]. The present study showed no significant association between IBS and eating regular meals per day, insufficient fiber in diet, daily tea and coffee consumption, cola intake or smoking. Karaman et al. observed that IBS prevalence was higher in individuals who did not eat regular meals and who had insufficient fiber in their diet. Also observed IBS prevalence increased as the daily consumption of cola increased, but did not find any relation between IBS and daily consumption of tea and coffee or with alcohol intake [13]. Laxative use was not common among our patient with IBS. Chronic laxative use usually exists in constipation- predominant patients [16]. Karaman et al. observed that laxative drug use was common among Patients with the most prevalent being constipation [13]. In the present study there was no significant correlation between IBS and previous abdominal operation. This finding is in contrast with finding by Hasler and Schoenfeld who reviewed the prevalence of abdominal and pelvic surgeries in IBS patients and reported an increased number of cholecystectomies, hysterectomies, appendectomies and other surgeries in IBS patients [29]. IBS are increased during and after sensorial tension and stress periods [30]. The current results showed a significant relation between IBS and previous psychological stress. Studies evaluating the role of acute stress have shown that stress can result in release of stress-related hormones that affect colonic sensorimotor function (eg, corticotropin-releasing factor (CRF) and inflammatory mediators (e.g., interleukin (IL)-1), leading to inflammation and altering GI motility and sensation [31]. However, other studies have shown no difference in psychiatric morbidity between IBS patients that improved and those that did not improve after 6–9 months follow-up [32].

Impact of IBS on quality of life: In a systematic review by Bijkerk et al, it was shown that the IBS-QOL is the best of the five IBS-specific QOL scores to establish changes in health-related QOL [33]. In the present study, patients with IBS had statistically significant lower scores for all IBS- QOL domains compared with the general population. With the lowest score of QOL that of the Food Avoidance scale (mean= 38.54, S.D.=27.33) and the highest score of QOL those of Relationships (mean= 69.79, S.D. =20.30) and sexual concerns (mean= 67.50, S.D =25.76). When the sexual concerns were assessed in our patients, it should be taken into account that they may hesitate or avoid expression of such topics even if they have any sexual problems. Cross-cultural difference between the countries (e.g. race, food, belief, social milieu and health-care system) might affect some dimensions of perception for the health-related QOL in patients with IBS. Kanazawa et al. observed that the mean overall score of the IBS-QOL-J in the Japanese subjects with IBS was significantly lower than those of the general population with the most affected domains are food avoidance (55.3 vs. 43.4), health worry (73.1 vs. 59.2) [34]. Other two studies showed a higher mean overall score of the IBS-QOL. In USA it was 63.2[14], while in Spain it was 75.5[35]. By logistic regression test, the daily cola consumption was unfortunately affecting QOL positively. IBS, Previous psychological stress, hard stool, straining, passing mucus and abdominal distention were negativity affecting QOL.

## Conclusion

IBS is a bio-psychosocial disorder. Biological, social, and psychological components play a role in disease perception, symptom generation, and healthcare seeking. For a physician to be truly effective in approaching the patient with IBS, all of these factors need to be considered. The results of the current study are important for directing our attention towards improving the quality of life of IBS patients in addition to symptomatic treatment.
